# A Conservative Approach by Unilateral Maximal Single-Muscle Recession Surgery for the Treatment of Cyclic Esotropia

**DOI:** 10.1155/2023/9402618

**Published:** 2023-01-16

**Authors:** Cem Evereklioglu, Fatih Horozoğlu, Hidayet Sener, Osman Ahmet Polat

**Affiliations:** Department of Ophthalmology, Division of Pediatric Ophthalmology & Strabismus, Erciyes University Medical Faculty, Kayseri, Turkey

## Abstract

**Purpose:**

Cyclic esotropia (alternate-day squint) is a poorly understood form of strabismus that mostly occurs at younger pediatric ages. It demonstrates classically a 48-hour cycle with 24 hours of manifest esotropia and 24 hours of orthotropia, which is conventionally managed by bilateral or unilateral 2-muscle surgery. We aimed to report a child with cyclic esotropia who was surgically treated by a conservative unilateral 1-muscle approach.

**Methods:**

Case report.

**Results:**

A 3.5-year-old girl presented to the strabismus department with an intermittent esodeviation for 2 years that became cyclic in the last 3 months. The diagnosis of primary classical cyclic esotropia was made after seeing her multiple times on different days. The girl was emmetropic bilaterally, had normal visual acuities in both eyes, and high-angle right esodeviation (45-50*^Δ^*) with normal laboratory and MRI results. Unilateral maximal single-muscle recession of the right medial rectus was performed, and the child was followed up for 9 months. The girl developed excellent alignment after the surgery both at distance and near without cyclic pattern, and near-normal stereopsis (by animals: 100 sec. of arc) with binocularity was reached. The girl did not experience under- or overcorrection nor have a recurrence postoperatively.

**Conclusion:**

This is the first report of “one eye single-muscle” surgery for high-angle cyclic esotropia. Conservative unilateral medial rectus recession seems to be sufficient to permanently block the circadian rhythm and restore binocular fusion and stereopsis.

## 1. Introduction

Cyclic esotropia is a mysterious phenomenon that most commonly occurs in early childhood and is characterized by a large angle of manifest but circadian esodeviation of the eye (crossed every other day) [1]. Classical cyclic esotropia has a 48-hour circadian rhythm of fixed orthophoria and esotropia that is associated with poor binocular function and suppression on “strabismic” days, though 24- to 96-hour cycles have also been described [2]. The conventional treatment is by two-muscle surgery, bimedial rectus muscle recession with or without posterior fixation sutures, or a unilateral recession/resection procedure according to the manifest deviation on the esotropic day [1].

## 2. Case Presentation

**Figure 1:**
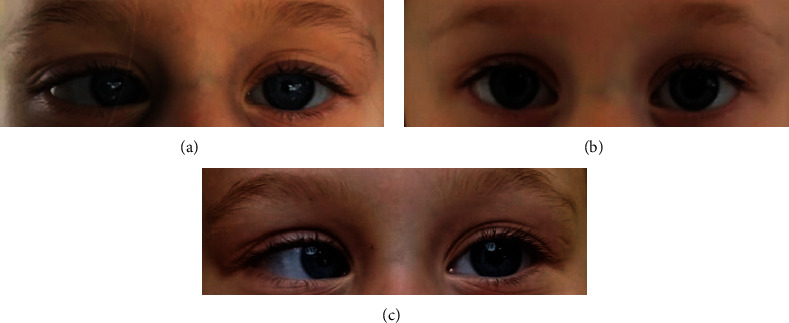
(a) A 3.5-year-old girl with right esotropia on the day of admission. (b) In the next day, the child is orthotropic. (c) She was again esotropic on the third scheduled “surgery” day.

A 3.5-year-old girl was seen in our clinic with the main complaint of intermittent convergent strabismus for 2 years that became cyclic in the last 3 months. The esotropia demonstrated a classical 48-hour cycle and showed circadian rhythm, appearing every alternating day without diplopia during the attack. The girl's and her family's past medical histories were unremarkable. The family of the child had kept a diary with many photographs on their cellphones that demonstrated right esotropia and orthotropia cycles day by day. The child was followed up for three consecutive days until the day of scheduled surgery. The girl had right esotropia on admission (Figure 1(a)), orthotropia on the second day (Figure 1(b)), and again right esotropia on the third “surgery” day (Figure 1(c)) with a diagnosis of “primary 48-hour/classical cyclic right esotropia.”

The girl's cycloplegic refraction revealed emmetropia with normal visual acuities bilaterally using E-test (20/20) both during the day of orthophoria and esotropia. Her eyes were orthotropic with near-normal stereoacuity on Titmus testing (by animals: 100 sec. of arc) on the “straight” day whereas the alternate prism cover test demonstrated a large right esotropia of 45-50*^Δ^* at both distance and near fixations on the “crossed” day with right suppression with no stereoacuity on Titmus testing. Ocular motility examination was full with no limitation, and all other neurological, laboratory, and MRI findings were found to be normal. Myasthenia gravis was ruled out and the child was not on any medication.

**Figure 2:**
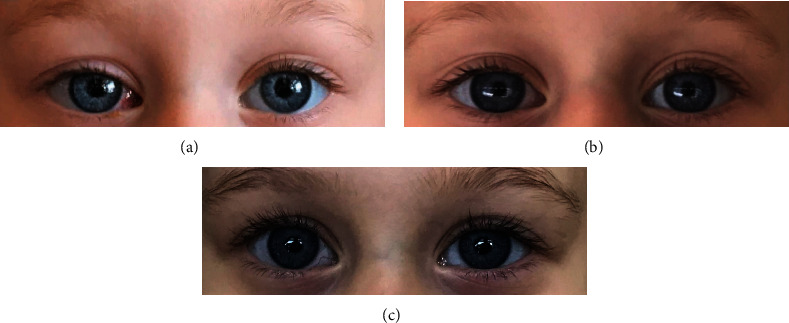
(a) Postoperative first-week photo with orthotropia. (b) The second photo at 6 months with no recurrence or over-/undercorrection. (c) The last photo at 9 months.

The mother of the girl was eager to have a unilateral operation for the deviated right eye only. In addition, maximal unilateral recession on the medial rectus was previously performed for noncyclic esodeviations [3]. Therefore, we decided to perform maximal “one eye single-muscle” surgery. Perioperatively, the insertion of the rectus muscle was seen to be normal, and the child underwent unilateral 7 mm recession of the right medial rectus. Postoperatively, the girl was seen at the first week (Figure 2(a)). The cycle was broken, the deviation disappeared, and the girl remained orthophoric afterward with full ocular movements without recurrence, six (Figure 2(b)) and ninth months postoperatively (Figure 2(c)). Postoperative lateral incomitance was not encountered, and permanent binocularity was established with positive stereoacuity on Titmus testing (by animals: 100 sec. of arc). Written informed consent was obtained from the family regarding the publication of the identifiable figures.

## 3. Discussion

Cyclic esotropia, characterized by periodic cycles of strictly repetitive “straight” and “crossed” eyes, is an extremely uncommon form of nonaccommodative convergent strabismus with an incidence between 1/3000 and 1/5000 and has been reported more commonly than cyclic exotropia or cyclic vertical deviations [1, 2]. Most cases occur spontaneously in childhood and are congenital but may be acquired after conditions that disrupt fusion or cause diplopia such as optic atrophy, ocular myositis, trauma, ocular or strabismic surgery, and CNS abnormalities [4].

The triggering basis for this phenomenon is unclear. However, dysfunction in the oculomotor nuclei or their interconnections, periodic sympathetic hyperactivity, cyclic oculomotor nerve dysfunction, and an adaptation to a peripheral afferent defect have been suggested in cyclic esotropia, though concomitant variations of behavioral rhythms (i.e., sleep-wake), have not been reported [1, 4, 5]. The periodic biological mechanism (i.e., body temperature and blood pressure) has been studied, but no explanation was made for the cyclic behavior of esotropia [5, 6].

Pediatric-onset cyclic esotropia begins around 3 years of age in the absence of any triggering event, and there seems to be no relation to visual acuity, accommodation, fatigue, illness, fusion, or binocular function for the beginning of a regular circadian rhythm. The patients demonstrate sufficient binocular single vision and improved stereoacuity during the orthotropic phase along with no symptoms of diplopia but poor or no stereopsis during the esotropic phase or as a result of microesotropia. As the girl in the present case had intermittent esotropia for 2 years, she demonstrated near-normal binocular vision both before and after the surgery. In addition, it is known that regular cyclicity of the esotropia during the previous three months indicates outstanding fusional potential.

Nonsurgical approaches by botulinum toxin injections to one or both medial rectus muscles may be an alternative way to permanently treat this unique disease. Indeed, an article demonstrated that 2 children with cyclic esotropia were successfully treated with a unilateral or bilateral single dose of botulinum toxin injection for at least 8 years of long-term follow-up [2]. So, botulinum toxin could be a conservative and predictable alternative way to break the cycle permanently, despite its limitations [7]. On the other hand, unilateral strabismus surgery may be an alternative choice for the permanent correction of cyclic esotropia, especially in cases with a high-angle deviation that is dosed for the angle of manifest strabismus. The traditional modality is either by 5.5-6.0 mm bimedial rectus muscle recession with or without posterior fixation sutures or by a unilateral recession/resection procedure based on the magnitude of the tropia measured on the esotropic phase [8]. Similarly, seven patients with cyclic esotropia underwent bilateral medial rectus recession, and all patients developed stable postoperative alignment in the long term with some degree of binocularity in five of six patients without recurrence in any patient [1]. Although a generally good outcome can be reached with bilateral muscle surgery or unilateral double-muscle procedures, overcorrection still exists in some pediatric and adult patients from cyclic esotropia to cyclic or noncyclic exotropia [1, 8]. Therefore, there is no classical treatment modality in these cases, and the management of such an interesting disease remains imprecise.

It seems that a conservative approach by choosing unilateral maximal “single-muscle” recession surgery only to the squinting eye may be sufficient not only to break the cyclic status of the disease but also to avoid possible consecutive exodeviation. In addition, the contralateral medial rectus or ipsilateral lateral rectus muscle can therefore be preserved for a possible undercorrection or late complete recurrence of large cyclic esotropia, which has previously been reported in patients with unilateral 5 mm recession/resection procedure or bimedial rectus muscle recession that required further corrective surgeries [8, 9]. This supports the theory that the underlying central disorder in cyclic strabismus is not affected by the peripheral manipulation of the extraocular rectus muscles, though the surgery still eradicates the situation intimately similar to removing the hands of the clock while leaving the clock ticking on [6]. Therefore, it seems that a conservative approach may be preferred, and therefore, some backup muscles can be preserved for possible future recurrence of cyclic esotropia.

As the manifest angle on the “crossed” phase is classically between 30*^Δ^* and 45*^Δ^*, the range for surgery is generally between 4 and 6 mm bimedial rectus muscle recession with or without posterior fixation suture [1, 10], indicating the lack of certainty about the approach. This raises the question of what is the least we can do to fix this kind of misalignment and still achieve successful results? We do not know the answer yet; however, it can be hypothesized that both unilateral single- or double-muscle surgery and bilateral double-muscle approach seem to be effective in blocking the circadian rhythm. The main limitation of this report is that there is no measurement of fusion either pre- or postoperatively. Therefore, large series are needed to see the results of this novel “unilateral single-muscle surgery” approach in such cases.

In conclusion, this is the first reported case of cyclic esotropia that was successfully treated by conservative “one eye single-muscle” recession surgery instead of classical bimedial rectus muscle recession or unilateral recession/resection surgery. In the present case, unilateral “single-muscle” surgery was found to be sufficient to block the cyclic nature of the manifest cyclic esotropia for the 9-month follow-up period, during which orthophoria was constantly maintained with good stereoacuity. Should a future possible recurrence be encountered, the surgeon will have a backup medial rectus muscle on the contralateral eye. However, future trials in large series are necessary to find an answer to the question “is a conservative approach by unilateral medial rectus recession, for instance, 5 mm or less effective to break the cycle?”, which is to be clarified.

## Data Availability

The patient's medical report is available.
